# Supplementary material for: U6 snRNA m6A modification is required for accurate and efficient cis- and trans-splicing of C. elegans mRNAs
Source: bioRxiv. 2023 Sep 16:2023.09.16.558044. Preprint. [Version 1] doi: 10.1101/2023.09.16.558044 (PMC10516052; doi:10.1101/2023.09.16.558044)
Supplement: Supplement 2 [file NIHPP2023.09.16.558044v1-supplement-2.pdf]

bioRxiv preprint doi: https://doi.org/10.1101/2023.09.16.558044; this version posted September 16, 2023. The copyright holder for this preprint (which was not certified by peer review) is the author/funder, who has granted bioRxiv a license to display the preprint in perpetuity. It is made available under aCC-BY 4.0 International license.

A

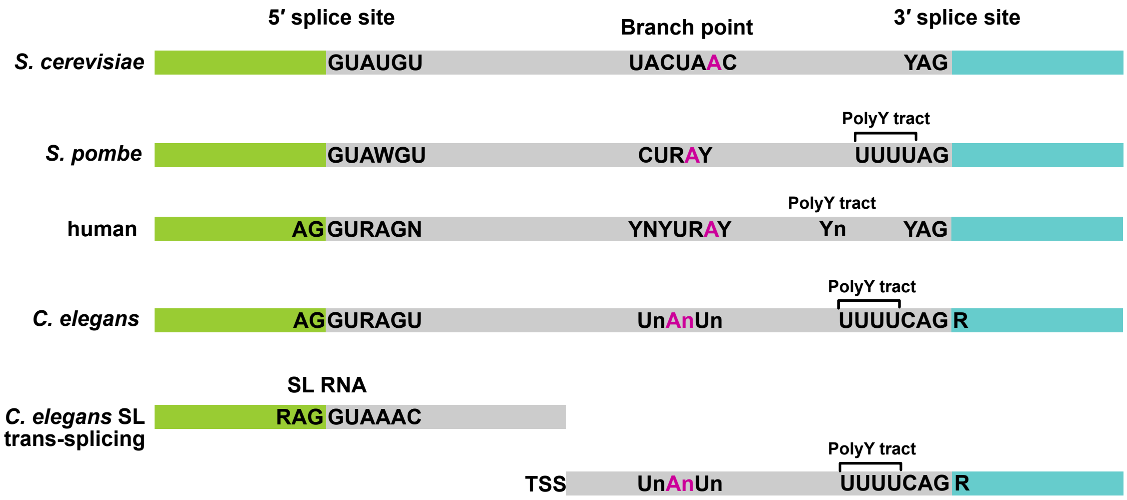

B

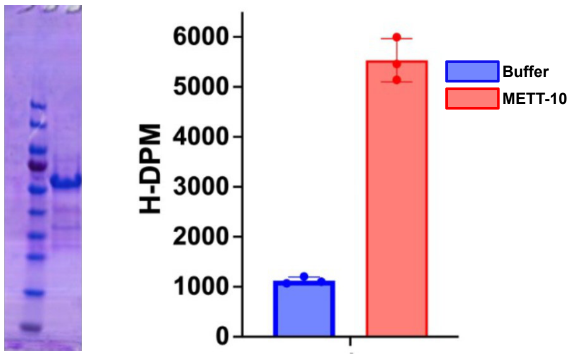

Figure S1.

Splice site motifs in different organisms and methylation of U6 by METT-10. **(A)** 5′SS, 3′SS and branch point sequences in *S. cerevisiae, S. pombe, humans, C. elegans and C. elegans* trans-splice sites. 5′SS exons are shown in green, 3′SS exons in turquoise and introns in grey. Y: pyrimidines. TSS: transcription start site. **(B)** Gel electrophoresis of recombinant purified METT-10 (left panel), used in *in vitro* methylation reaction of U6 snRNA (right panel).

bioRxiv preprint doi: https://doi.org/10.1101/2023.09.16.558044; this version posted September 16, 2023. The copyright holder for this preprint (which was not certified by peer review) is the author/funder, who has granted bioRxiv a license to display the preprint in perpetuity. It is made available under aCC-BY 4.0 International license.

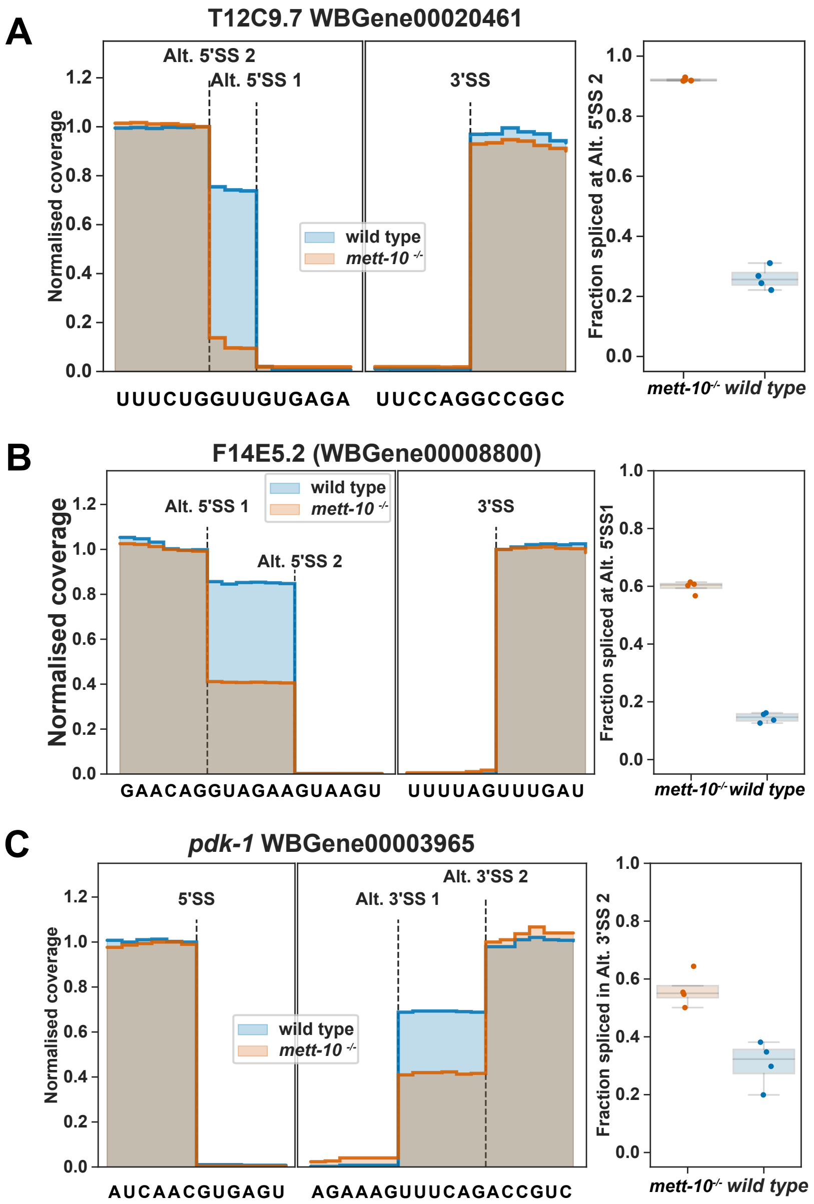

Figure S2.

Additional examples of alternative 5′SS and 3′SS usage events. **(A)** T12C9.7 encodes the mitotic specific cyclin B2 and in wild type animals more frequently spliced at the Alt. 5′SS 1 //GUGAG. In *mett-10*$^{-/-}$, most of the splicing shifts to Alt. 5′SS 2 UG//GUUGU. **(B)** F14E5.2 encodes the *C. elegans* orthologue of the human GLG1 and F14E5.2 exon 8 is most frequently spliced at Alt. 5′SS 2 AA//GUAAG in wild-type animals. In *mett-10*$^{-/-}$ animals, 5′SS choice moves to the nearby Alt. 5′SS 1 AG//GUAGA. **(C)** *pdk-1* is the *C. elegans* orthologue of the human cancer associated kinase PDPK1. *Pdk-1* is frequently spliced in wild-type animals at both Alt. 3′SS 1 and Alt. 3′SS 2. In *mett-10*$^{-/-}$ animals most of the splicing events move to the Alt. 3′SS 2.

bioRxiv preprint doi: https://doi.org/10.1101/2023.09.16.558044; this version posted September 16, 2023. The copyright holder for this preprint (which was not certified by peer review) is the author/funder, who has granted bioRxiv a license to display the preprint in perpetuity. It is made available under aCC-BY 4.0 International license.

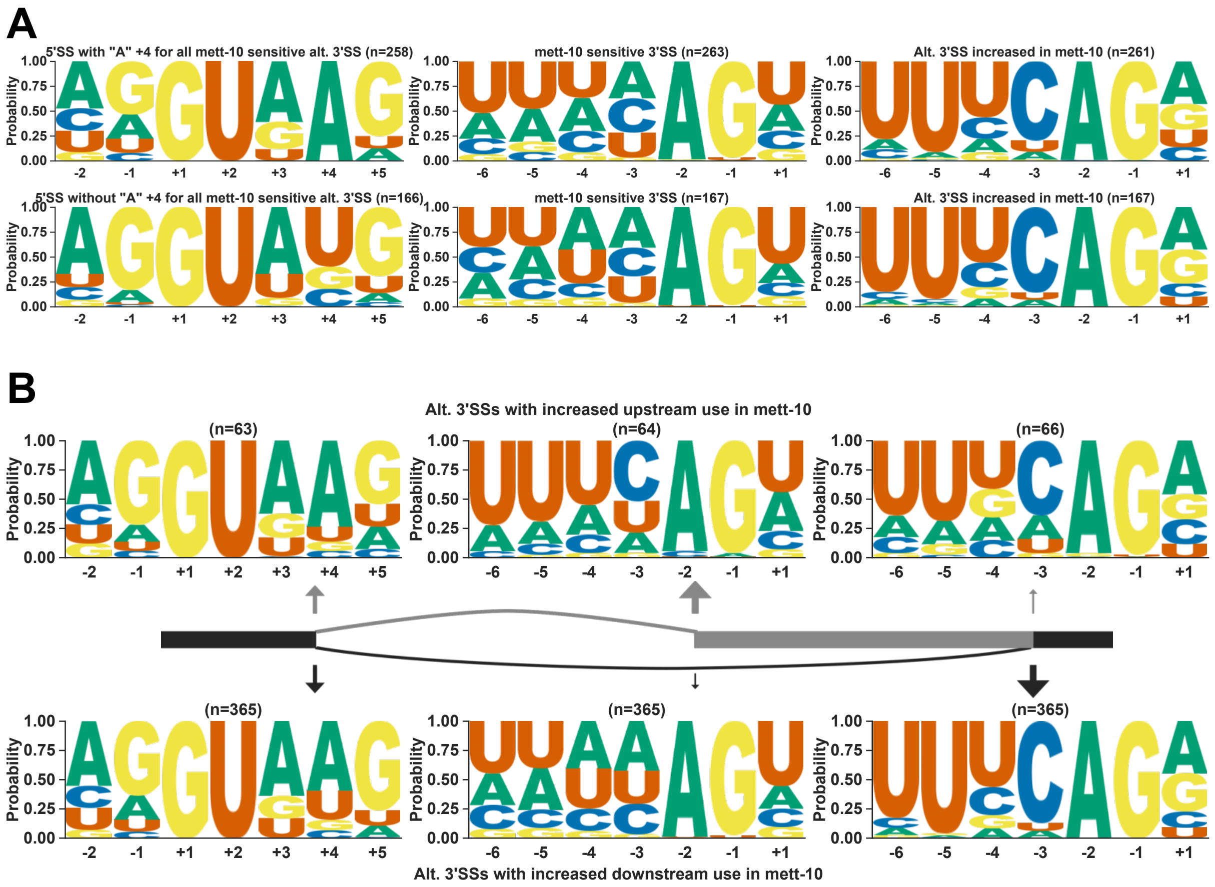

Figure S3.

Alternative 3′SS usage. **(A)** Most alternative 3′SS events are associated with 5′SSs with +4A (263) as opposed to 5′SSs without +4A, which are enriched for AG//GU (167). *mett-10*$^{-/-}$ sensitive 3′SSs have weak 3′SS motif of UUUCAG/R and there is a switch from these 3′SSs to a strong 3′SS with a clear UUUCAG/R motif in *mett-10*$^{-/-}$. **(B)** Majority of *mett-10*$^{-/-}$ sensitive 3′SS events (365) shift downstream to a stronger 3′SS motif (bottom panel). Alternative 3′SS events that shift upstream are limited (64) and tend to shift to a weaker 3′SS motif, although the difference is less clear.

bioRxiv preprint doi: https://doi.org/10.1101/2023.09.16.558044; this version posted September 16, 2023. The copyright holder for this preprint (which was not certified by peer review) is the author/funder, who has granted bioRxiv a license to display the preprint in perpetuity. It is made available under aCC-BY 4.0 International license.

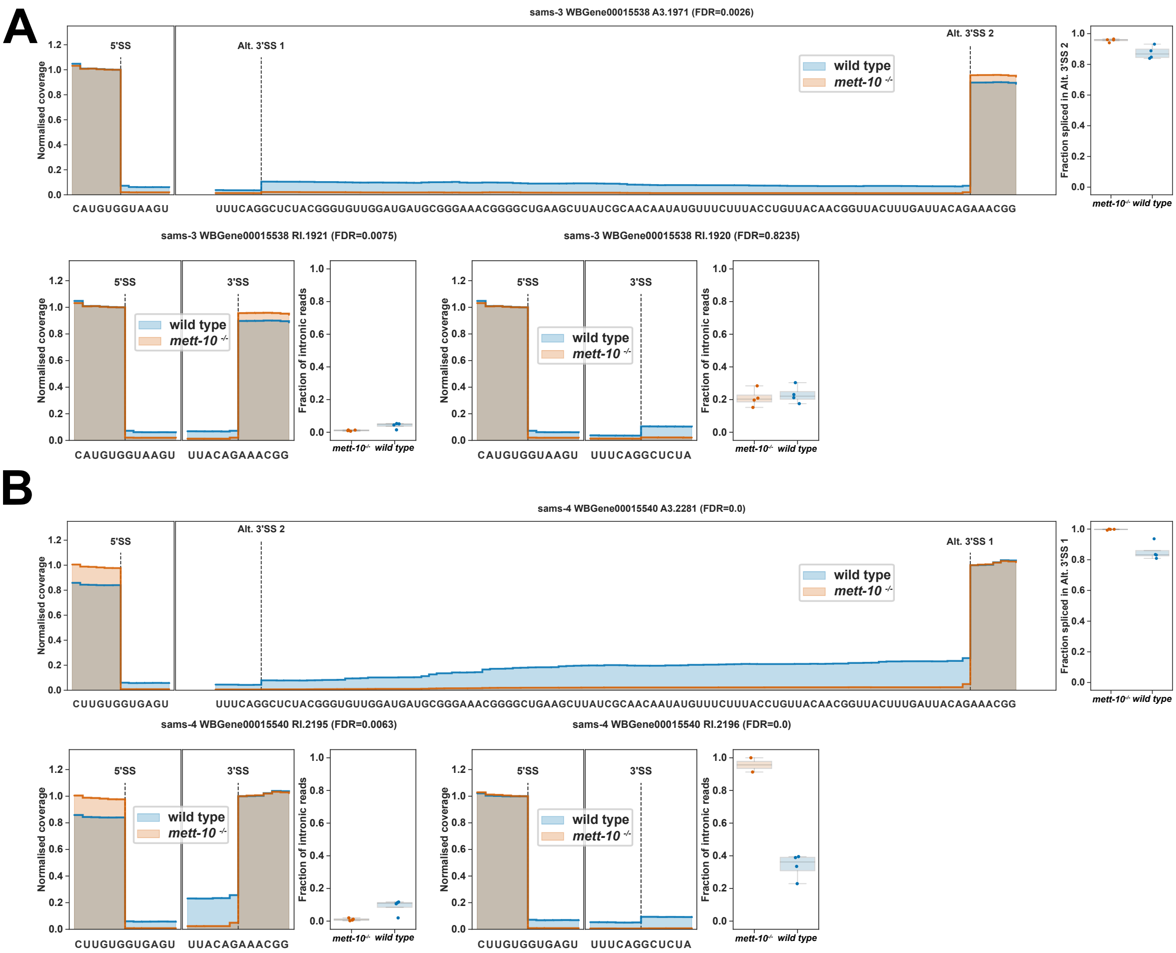

Figure S4.

sams gene alternative splicing events. **(A)** RNA-Seq coverage of *sams-3* gene alternative splicing events for alternative 3′SS usage and two different intron retention events. **(B)** RNA-Seq coverage of *sams-4* gene alternative splicing events for alternative 3′SS usage and two different intron retention events.

bioRxiv preprint doi: https://doi.org/10.1101/2023.09.16.558044; this version posted September 16, 2023. The copyright holder for this preprint (which was not certified by peer review) is the author/funder, who has granted bioRxiv a license to display the preprint in perpetuity. It is made available under aCC-BY 4.0 International license.

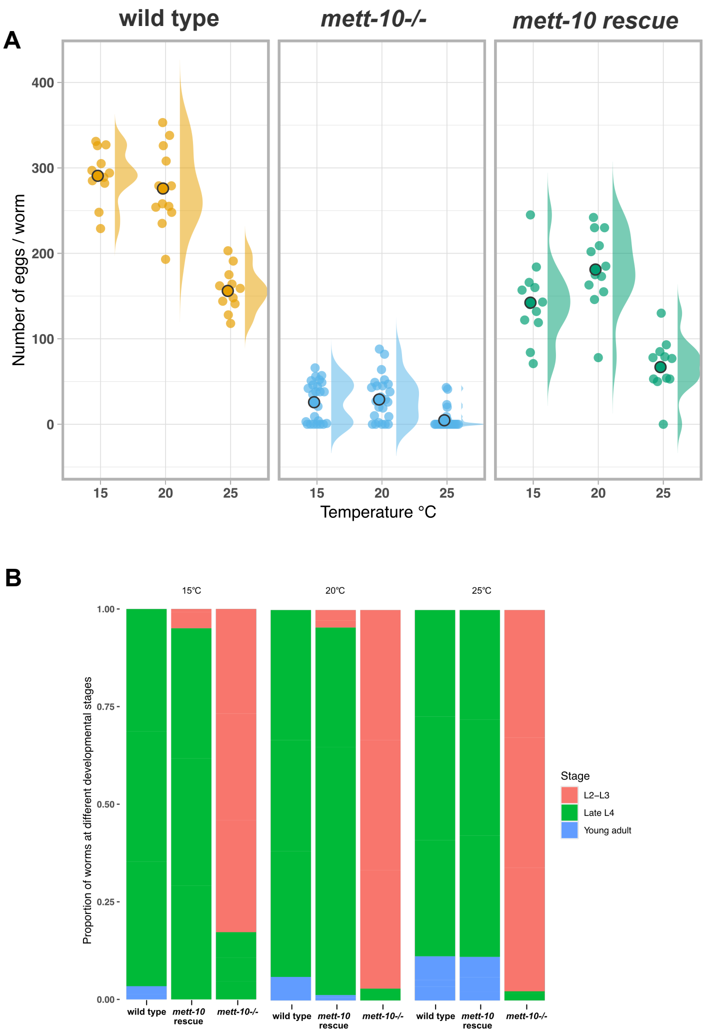

Figure S5.

Phenotypic rescue of *mett-10(null)* animals with a germline expressed *mett-10* transgene. **(A)** Animals expressing the *mett-10 rescue* transgene in the germline and in a *mett-10-/-* background have higher progeny per animal at all 3 temperatures tested. **(B)** Animals expressing the *mett-10 rescue* transgene in the germline and in a *mett-10-/-* background have developmental timing similar to wild-type animals.

bioRxiv preprint doi: https://doi.org/10.1101/2023.09.16.558044; this version posted September 16, 2023. The copyright holder for this preprint (which was not certified by peer review) is the author/funder, who has granted bioRxiv a license to display the preprint in perpetuity. It is made available under aCC-BY 4.0 International license.

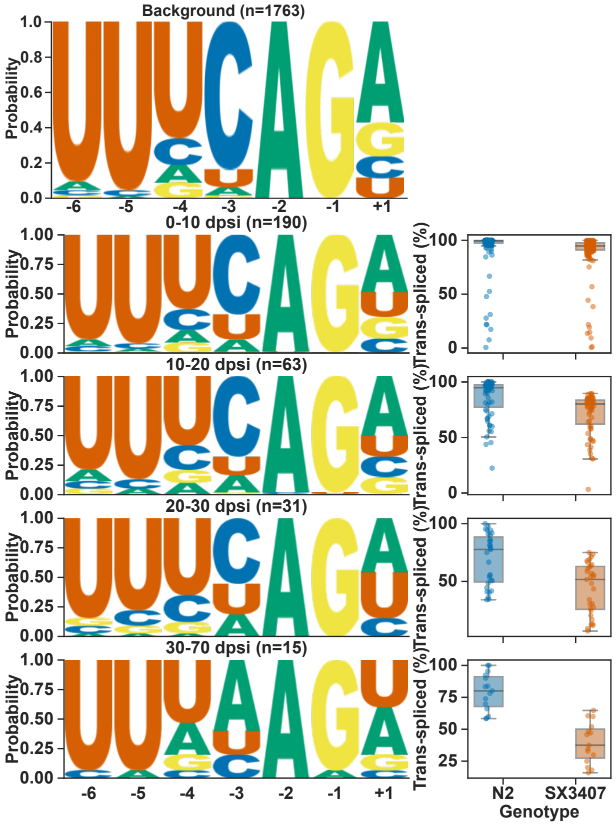

Figure S6.

Analysis of 3′ trans-splice site motifs among transcripts with different levels of trans-splicing defects as in Figure 6A.

bioRxiv preprint doi: https://doi.org/10.1101/2023.09.16.558044; this version posted September 16, 2023. The copyright holder for this preprint (which was not certified by peer review) is the author/funder, who has granted bioRxiv a license to display the preprint in perpetuity. It is made available under aCC-BY 4.0 International license.

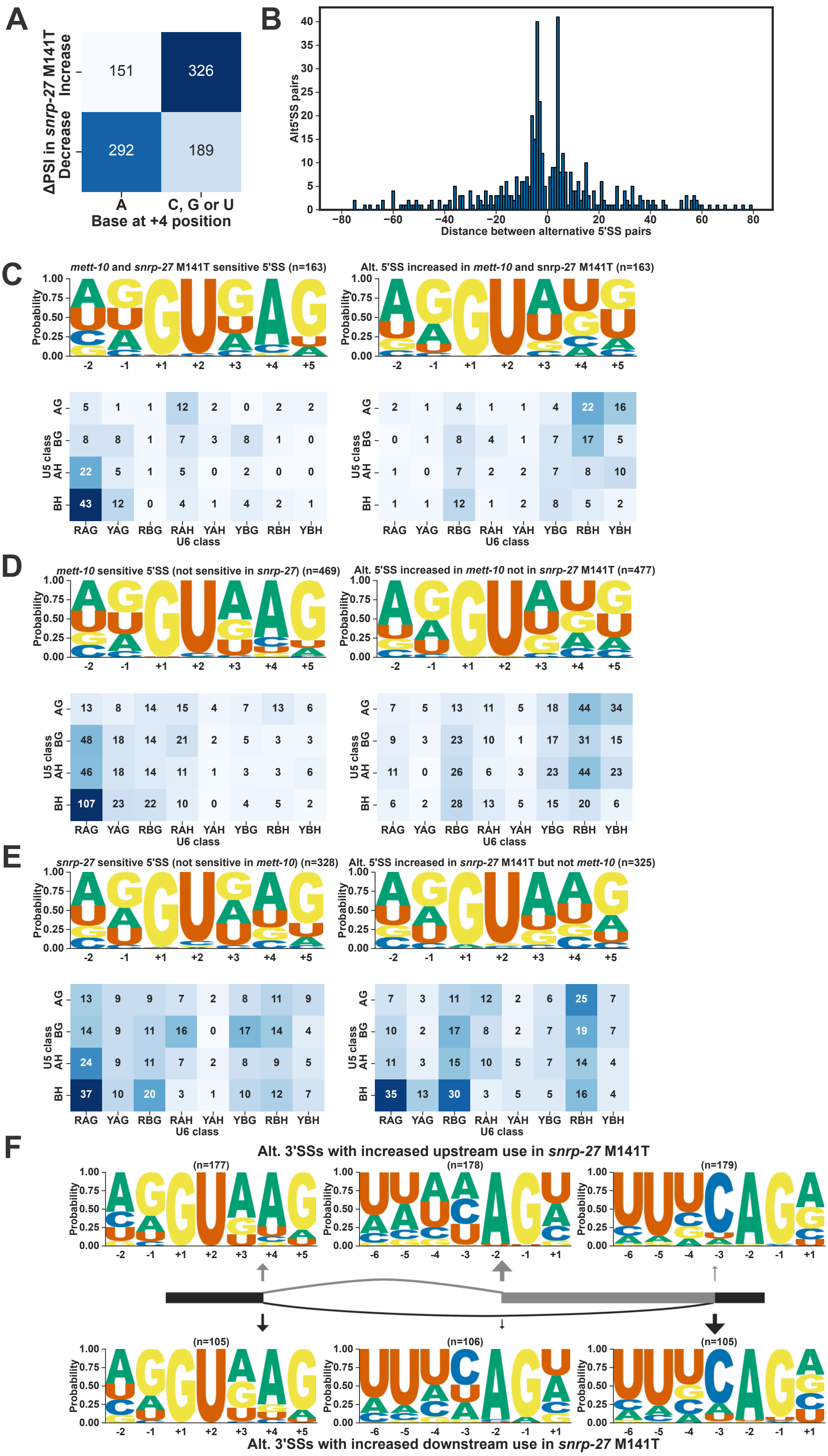

bioRxiv preprint doi: https://doi.org/10.1101/2023.09.16.558044; this version posted September 16, 2023. The copyright holder for this preprint (which was not certified by peer review) is the author/funder, who has granted bioRxiv a license to display the preprint in perpetuity. It is made available under aCC-BY 4.0 International license.

Figure S7.

SNRP-27 is required for effective cis-splicing. **(A)** Heat-map showing presence or absence of +4A for *snrp-27* sensitive 5′SS events **(B)** Histogram of distance between alternative 5′SS pairs and the number of events. **(C, D and E)** 5′SS sequence motif and the frequency U5 and U6 interacting sequences for 5′SSs sensitive to **(C)** both *mett-10* and *snrp-27*, **(D)** only to *mett-10* and **(E)** only to *snrp-27.* **(F)** Sequence motif 3′SSs that are sensitive to *snrp-27* and the alternative 3′SS usage either shifts upstream (upper panel) or downstream (bottom panel).

bioRxiv preprint doi: https://doi.org/10.1101/2023.09.16.558044; this version posted September 16, 2023. The copyright holder for this preprint (which was not certified by peer review) is the author/funder, who has granted bioRxiv a license to display the preprint in perpetuity. It is made available under aCC-BY 4.0 International license.

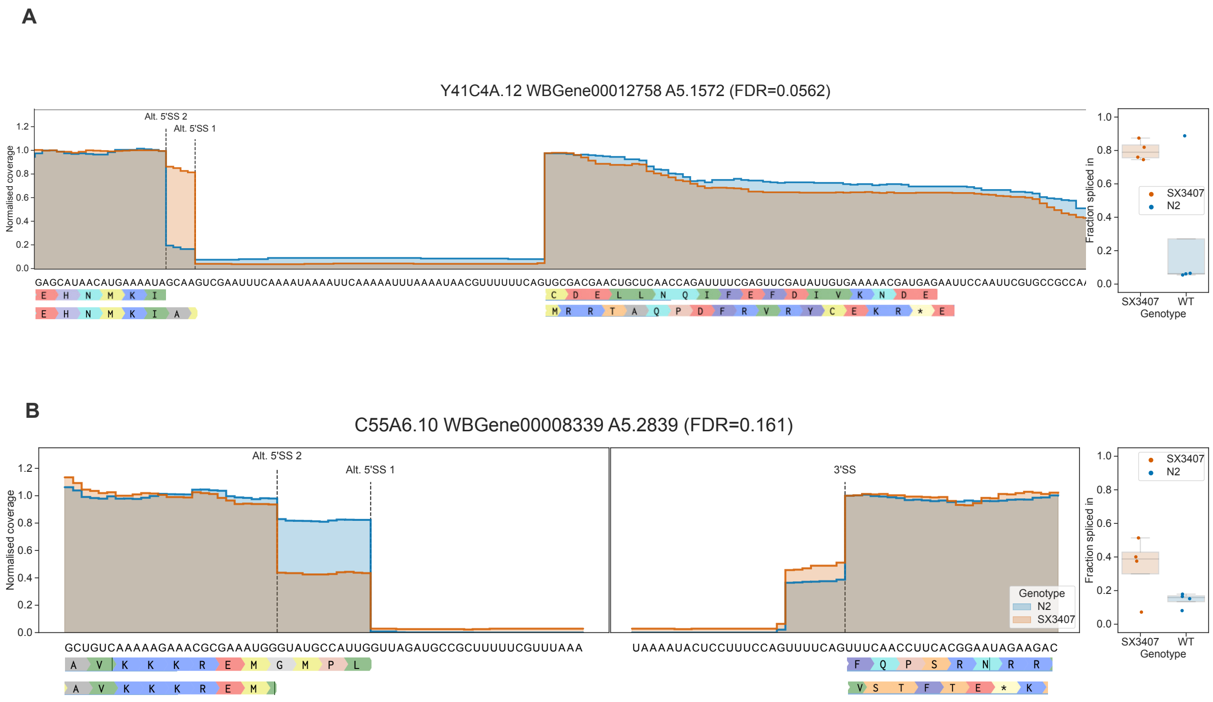

Figure S8.

Examples of genes where alternative 5′SS usage generates a frame-shift in the open reading frame of **(A)** Y41C4A.12 and **(B)** C55A6.10.

bioRxiv preprint doi: https://doi.org/10.1101/2023.09.16.558044; this version posted September 16, 2023. The copyright holder for this preprint (which was not certified by peer review) is the author/funder, who has granted bioRxiv a license to display the preprint in perpetuity. It is made available under aCC-BY 4.0 International license.

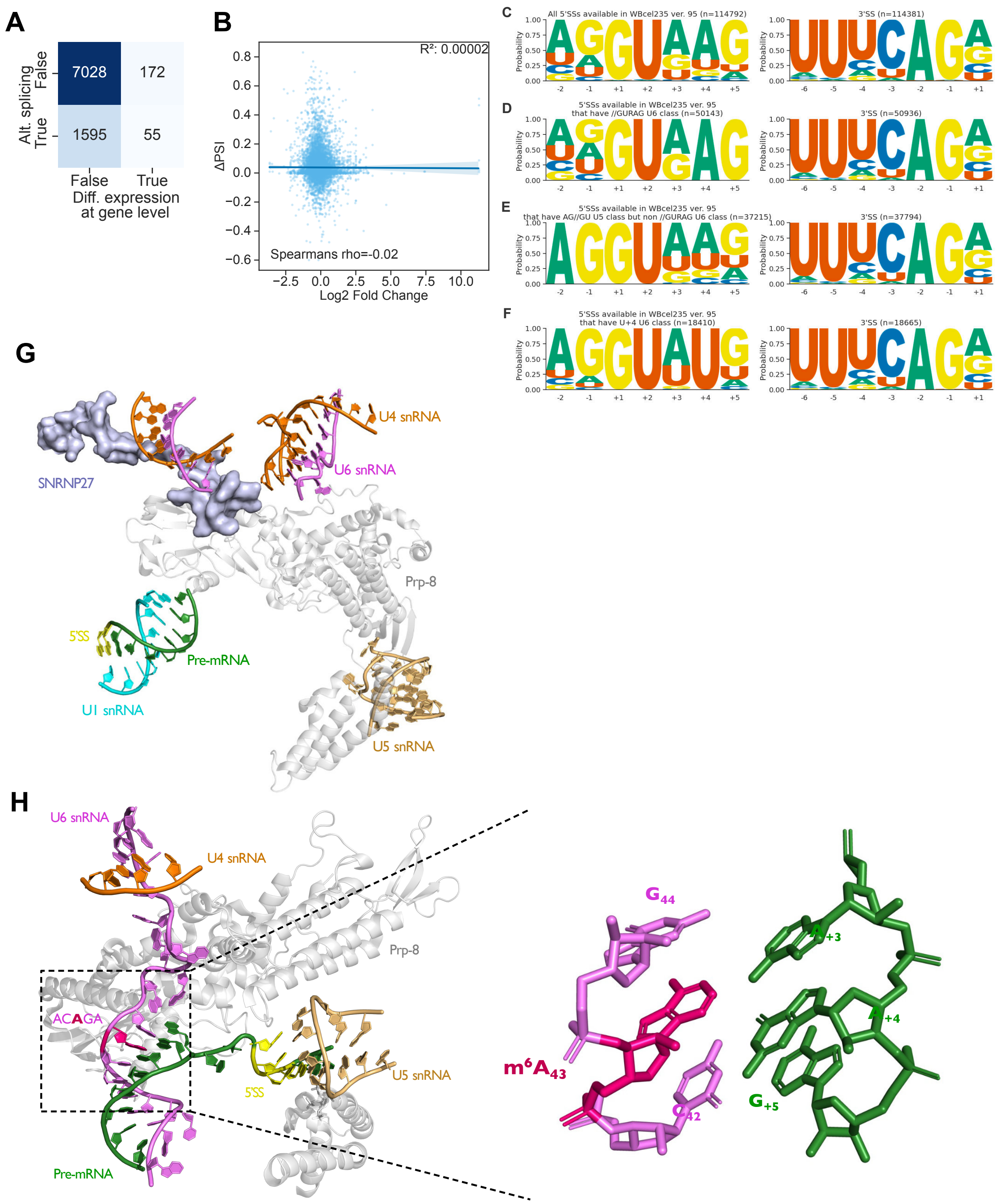

Figure S9.

mett-10 sensitive splicing changes do not correlate with the gene expression changes, sequence motifs of diverse splice sites in *C. elegans* and cryo-EM structures of pre-B and B complexes. **(A)** Heat map showing the overlap of genes with significant change in their expression and splicing. Only 55 genes with an FDR < 0.05 have a change in splicing and

bioRxiv preprint doi: https://doi.org/10.1101/2023.09.16.558044; this version posted September 16, 2023. The copyright holder for this preprint (which was not certified by peer review) is the author/funder, who has granted bioRxiv a license to display the preprint in perpetuity. It is made available under aCC-BY 4.0 International license.

expression. **(B)** Correlation of splicing changes in *mett-10* mutants animals compared to wild type (ΔPSI) and the log2 fold change values. **(C-F)** Sequence motif of 5′SSs and 3′SSs for **(C)** all *C. elegans* genes, **(D)** with a //GURAG motif, **(E)** AG//GU and not //GURAG and **(F)** +4U only. **(G)** cryo-EM structure of the pre-B complex showing SNRNP27K, U6 snRNA, U1 snRNA, U5 snRNA and pre-mRNA PDB: 6QX9. **(H)** cryo-EM structure of the B-complex showing U6 snRNA - pre-mRNA 5′SS interactions PDB: 6AHD.
